# Impactful disease research: a DMM year in review

**DOI:** 10.1242/dmm.050098

**Published:** 2023-01-31

**Authors:** Rachel Hackett, E. Elizabeth Patton

**Affiliations:** ^1^ The Company of Biologists, Bidder Building, Station Road, Histon, Cambridge CB24 9LF, UK; ^2^ MRC Human Genetics Unit, and Edinburgh Cancer Research, CRUK Scotland Centre, Institute of Genetics and Cancer, The University of Edinburgh, Western General Hospital, Crewe Road South, Edinburgh EH4 2XU, UK

## Abstract

**Summary:** Editor-in-Chief Liz Patton reflects on the achievements of DMM and looks to the future of the journal.

It is a pleasure to start the new year by reflecting on the year that has passed at Disease Models & Mechanisms (DMM) and looking forward to the months ahead. In reviewing 2022, it is encouraging to see how the DMM community is starting to come back together following the worst months of the ongoing pandemic, which we know deeply affected many of us in our community and continues to challenge us.

One area that has been especially wonderful to see re-flourish is our Travelling Fellowships and Conference Travel Grants. These are now more popular than ever, with DMM Editors assessing record numbers of applications from all around the world. Happily, the Board of Directors of The Company of Biologists recently made the decision to increase the funding available, meaning that DMM can support more early-career researchers in developing their careers through a collaborative visit to another laboratory or attending a conference relevant to DMM.

Looking forward, we are excited to present the third iteration of the DMM Journal Meeting – ‘Infectious Diseases Through an Evolutionary Lens’ – organised by Wendy Barclay, Sara Cherry, DMM Editor David Tobin and Russell Vance. This meeting will bring together leading experts in infectious diseases, host–pathogen interactions and evolutionary biology to examine new insights into infectious diseases, including pathogen evolution and emergence, and their treatment. We have a great venue in BMA (British Medical Association) House in London, and our organisers have recruited a fabulous line-up of speakers (Box 1). Registration is opening in February; we hope to see you there for what promises to be a fascinating and timely meeting.

**Box 1. DMM Journal Meeting – Infectious Diseases Through an Evolutionary Lens**

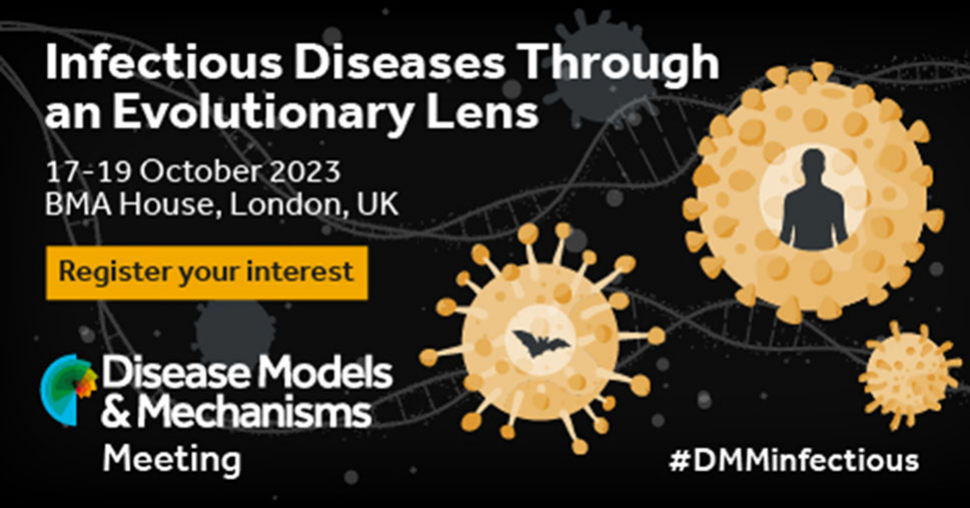

**Organisers:**

**Wendy Barclay**, Imperial College London, UK

**Sara Cherry**, University of Pennsylvania, USA

**David Tobin**, Duke University, USA

**Russell Vance**, University of California, Berkeley, USA

**Speakers:**

**Judi Allen**, The University of Manchester, UK

**Alfred Amambua-Ngwa**, Medical Research Council Unit The Gambia at

the London School of Hygiene & Tropical Medicine, The Gambia

**Sonja Best**, National Institute of Allergy and Infectious Diseases, USA

**Heran Darwin**, New York University, USA

**Nels Elde**, The University of Utah, USA

**Andrea Gamarnik**, Fundación Instituto Leloir, Argentina

**Philip Kranzusch**, Harvard University, USA

**Johannes Krause**, Max Planck Institute for Evolutionary Anthropology,

Germany

**Brenda Kwambana-Adams**, Liverpool School of Tropical Medicine, UK

**Harmit Malik**, Fred Hutchinson Cancer Center, USA

**João Marques**, Federal University of Minas Gerais, Brazil

**Lalita Ramakrishnan**, University of Cambridge, UK

**Stephen Russell**, Mayo Clinic, USA

**Vanessa Sancho-Shimizu**, Imperial College London, UK

**Paul Sharp**, The University of Edinburgh, UK

**Tyler Starr**, Fred Hutchinson Cancer Center, USA

**Sarah Tishkoff**, University of Pennsylvania, USA

**Emily Troemel**, University of California, San Diego, USA

**Linfa Wang**, Duke-NUS Medical School, Singapore

**Katherine Wu**, The Atlantic, USA

We have already started planning our 2024 DMM Journal Meeting, hosted at the Institute of Genetics & Cancer in Edinburgh, alongside Wendy Bickmore, Luke Boulter, Sara Brown, Pleasantine Mill and DMM Editor Owen Sansom. The meeting’s focus will be ‘Pre-clinical modelling of human genetic disease and therapy’. Speakers will discuss advances in modelling human genetic disease, and the latest technologies and challenges associated with generating relevant human genetic mutations in multi-modal systems, including advanced complex cellular models, model organisms and large-animal models. The meeting will feature a panel discussion with clinicians and patients engaged in gene-editing clinical trials. Rapid advances in gene editing have revolutionised our ability to model human genetic disease and provided new hope for gene-editing therapies, and we are excited to engage with the DMM community for this important topic.

We have been thrilled to see our vision for the front section (invited review-type material) of DMM come to fruition (Patton, 2021). Regular readers of DMM will have noticed that we have made a concerted effort to ensure that each DMM issue has an Editorial that is topical and interesting to our disease biology community, covering subjects ranging from mouse and *Drosophila* models of human disease (Clapcote, 2022; Sansom, 2022; Verheyen, 2022) to infectious disease (Tobin, 2022; Mostowy, 2022) and the predictive power of models of neuromuscular disease (van Putten, 2022). Our newly launched Perspective pieces have flourished this year. These opinionated pieces about a specific disease or challenge include recommendations from The Jackson Laboratory on improving biomedical research using animals (Svenson et al., 2022), a discussion on how variation in mouse models is central to improving translatability of pre-clinical research (Devlin and Roberts, 2022) and perspectives on how inducing autophagy can promote healthy ageing (Zinecker and Simon, 2022). And our long-established Review and At a Glance posters continue to go from strength to strength. Given that DMM is fully Open Access, all these articles are free to read and reuse, providing everyone with a valuable resource for teaching, conferences and seminars.

DMM holds regular meetings of its Editor team; we always aim to identify important areas of focus for DMM. Following one such discussion, we commissioned a subject focus on ‘Genetic variance in human disease’ (see image; Lek et al., 2022). Working closely with DMM Editor Monkol Lek, this ongoing series of invited Interviews, Perspectives and Reviews reflects on the latest advances in our understanding of genetic variance and genotype–phenotype complexities in disease, including using computational and deep mutational scanning to interpret protein variant effects (Livesey and Marsh, 2022) and the role of *ALDH2* variance in disease and global populations (Chen et al., 2022).

**Figure:**
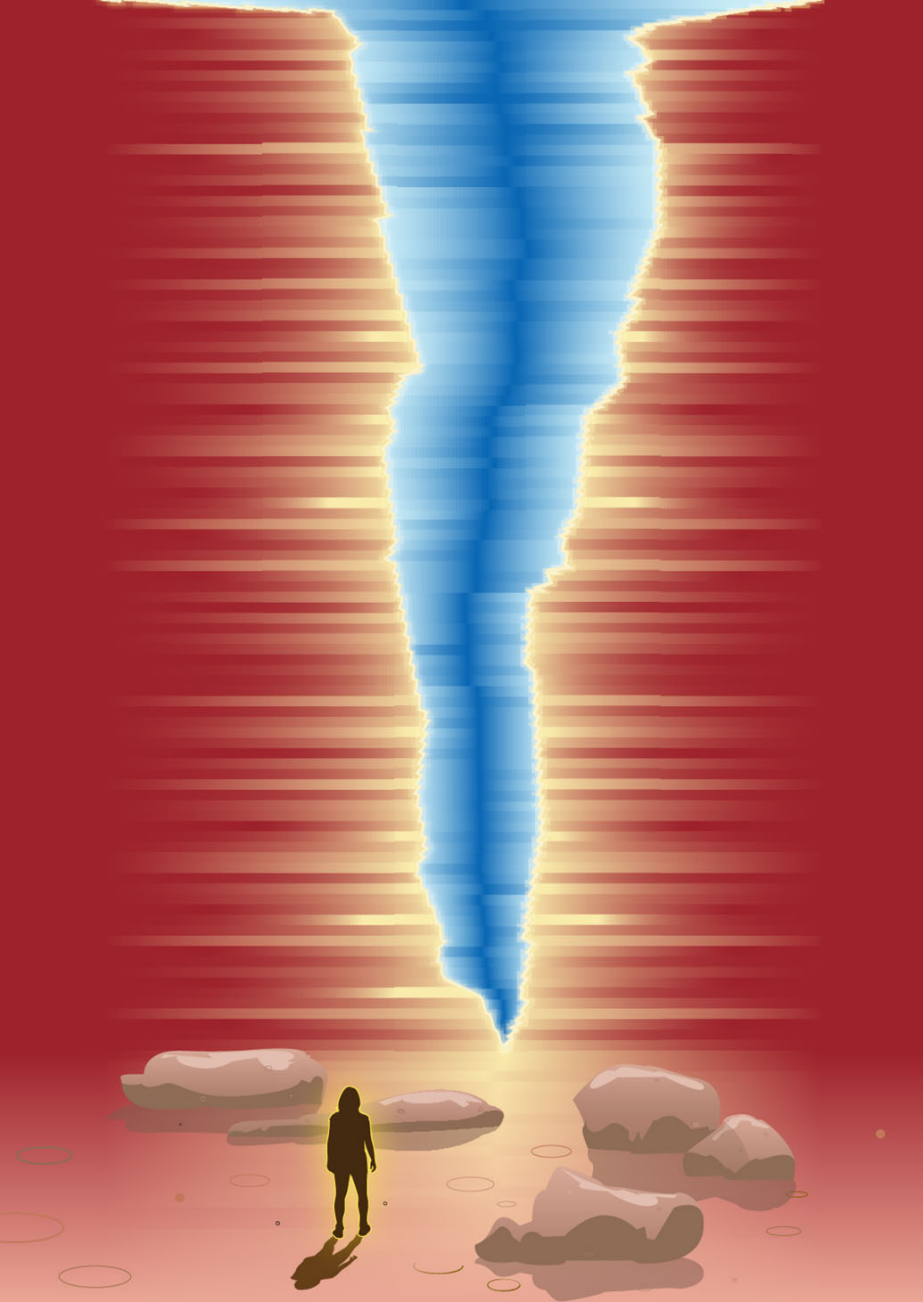


DMM always enjoys celebrating landmarks and events relevant to disease biology. For example, in December 2022, we were delighted to mark the 200th anniversary of the birth of Louis Pasteur with an Editorial from Serge Mostowy (Mostowy, 2022) and A Model for Life interview with Pascale Cossart (Cossart, 2022).

Our A Model for Life interviews are always a fascinating read (or watch). These voices of our community give a unique perspective on a life in disease biology research. Recent interviews include Pascal Cossart, Hugo Bellen, Jyoti Joshi and Monkol Lek, plus Ross Cagan interviewing Kevan Shokat on targeting RAS. We were thrilled with Joan Heath's interview of Joan Steitz and Suzanne Cory, prompted by the occasion of International Women's Day. Please take a moment to watch some of the associated videos, which are special to share with trainees. As a new initiative in 2023, and working with DMM Editor and clinician scientist James Amatruda, we are conducting interviews with patient advocates, and look forward to hearing the perspectives of people with lived experiences of disease.

Each year, DMM collates a new subject collection, launching with a dedicated Special Issue. In February 2022, we published our latest Special Issue – The RAS Pathway: Diseases, Therapeutics and Beyond – guest edited by Donita Brady and Arvin Dar. The collection includes Research, Resource and Review-type articles showcasing RAS-driven mechanisms of disease progression, as well as approaches to treat and modify the disease course (Anastasaki et al., 2022; Redding et al., 2022; Kamata et al., 2022; Inubushi et al., 2022). It also features an important Perspective from Katherine Rauen, outlining the key principles to defining RASopathies in patients (Rauen, 2022).

We have also begun compiling articles for our next Special Issue – Moving Heart Failure to Heart Success: Mechanisms, Regeneration & Therapy. This issue is being coordinated by guest editors Jeroen Bakkers (Hubrecht Institute, The Netherlands), Milena Bellin (University of Padova, Italy, and Leiden University Medical Center, The Netherlands) and Ravi Karra (Duke University School of Medicine, USA). Articles will focus on the dysregulation of pathways, disease progression and approaches to treat and modify the course of heart failure using *in vitro* and *in vivo* model systems.

As outlined in a previous Editorial, quality disease research and accessibility are the twin pillars of DMM. In January 2022, we published an Editorial outlining the ways in which we, and The Company of Biologists, are working towards enhancing the accessibility of DMM (Hackett and Patton, 2022). From 2023, DMM is included in more of the Read & Publish agreements offered by The Company of Biologists. This enables discounted or fee-free publication of an uncapped number of Research and Resource articles in DMM for corresponding authors at participating institutions, including the University of California, Max Planck Institutes, Monash University, The University of Edinburgh, University of Cambridge, National Institutes of Health Institutes, Johns Hopkins University and the Francis Crick Institute. Find out whether your institute is included here and whether you can publish in DMM for free!

When considering the other pillar – quality disease research – we have focused recently on our Resource articles. We often receive submissions that present a new model, methodology, datasets and/or technique that are important for the disease modelling community; these are generally most appropriate to be published as a Resource article. We actively encourage their submission and have honed our description of these in our information for authors. To this end, starting with articles published in 2022, we are excited to present a new Outstanding Resource Article Prize (£1000) to the first author of the Resource article judged by the Editors to be the exceptional article of its type. This is in addition to the existing Outstanding Research Article Prize (£1000). Establishing a separate award for Resource articles allows us to showcase some of the articles we publish that significantly contribute to the progression of disease modelling technologies and research (find out more in a recent Editorial; Hooper and Hmeljak, 2022).

Our Resource articles, as well as our Research articles and longer front section pieces, are reviewed by experts in the field. We would like to take the opportunity, as we do at the start of every year, to thank our reviewers for their time, expertise and dedication. The names of our 2022 reviewers, including their co-reviewers, are listed in the supplementary information. We also thank those reviewers of articles transferred to DMM from Review Commons.

Finally, we thank all our readers and authors for supporting DMM. As always, we appreciate hearing from you and value any feedback. We look forward to continuing and growing our personal connections in the coming year, and wish you all the very best for 2023.

## Supplementary Material

10.1242/dmm.050098_sup1 Supplementary information
