# Supplementary material for: Impactful disease research: a DMM year in review
Source: Dis Model Mech. 2023 Jan 31;16(1):dmm050098. doi: 10.1242/dmm.050098 (PMC9922873; doi:10.1242/dmm.050098)
Supplement: Supplementary information [file dmm-16-050098-s1.pdf]

Reviewers for Disease Models & Mechanisms 2022

Catherine Abbott, University of Edinburgh, UK

Helen Abud, Monash University, Australia

Swarnali Acharyya, Columbia University, USA

Johannes Aerts, Leiden University, the Netherlands

Devika Agarwal, Weatherall Institute Of Molecular Medicine, Oxford, UK

Pankaj Agrawal, Boston Children's Hospital and Harvard Medical School, USA

Imran Ahmad, The Beatson Institute for Cancer Research, UK

Maria Akhmanova, Institut of Science and Technology, Austria

Tomas Aleman, University of Pennsylvania, USA

Mosab Ali, University of Oxford, UK

Gianfranco Alpini, Indiana University School of Medicine, USA

James Alspaugh, Duke University School of Medicine, USA

Jeffrey Amack, SUNY Upstate Medical University, USA

James Amatruda, Children's Hospital Los Angeles, USA

Sai Balaji Andugulapati, CSIR-Indian Institute of Chemical Technology, India

Jose Aponte, University of Calgary, Canada

Brock Arivett, UAB School of Medicine, USA

Paul Armstrong, University of Leeds, UK

Duchon Arnaud, CNRS, France

Jun Aruga, Nagasaki University, Japan

Sophie Astrof, Rutgers University, USA

Adam Avery, Oakland University, USA

Wolfgang Baehr, University of Utah, USA

Richard Bagnall, Centenary Institute, Australia

Hua Bai, Iowa State University, USA

Sabine Bailly, Laboratoire Biologie du Cancer et de l'Infection, France

Sheila Baker, University of Iowa, USA

Jeroen Bakkers, Hubrecht Institute, the Netherlands

Angela Ballantyne, University of Otago, New Zealand

Erdem Bangi, Florida State University, USA

Nicholas Barker, Institute of Molecular and Cell Biology, Singapore

Sami Barmada, University of Michigan, USA

Serena Barral, University College London, UK

Sebastian Bass-Stringer, Murdoch Children's Research Institute, Australia

Tyler Beames, University of Wisconsin-Madison, USA

Jean-François Beaulieu, Université de Sherbrooke, Canada

Richard Behringer, MD Anderson Cancer Center, USA

David Beier, University of Washington, USA

David Bennett, University of Oxford, UK

Yehuda Ben-Shahar, Washington University in St. Louis, USA

Jason Berman, University of Ottawa, Canada

Andreia Bernardo, The Francis Crick Institute, UK

Jimena Berni, University of Sussex, UK

Akshay Bhinge, University of Exeter, UK

Sanjay Bidichandani, University of Oklahoma Health Sciences Center, USA

Caroline Binda, Cardiff University, UK

Colin Bingle, University of Sheffield Medical School, UK

Thomas Bird, CRUK Beatson Institute, UK

Oliver Blacque, University College Dublin, Ireland

Albert Blanch Asensio, Leiden University Medical Center, the Netherlands

Anthony Bleyer, Wake Forest Baptist Medical Center, USA

Karen Blyth, Beatson institute, UK

Rolf Bodmer, Sanford Burnham Prebys Medical Discovery Institute, USA

Benjamin Bolker, McMaster University, Canada

Paul Bollyky, Stanford University, USA

Nadège Bondurand, Imagine Institute, France

Laura Borodinsky, UC Davis Cancer Center, USA

Alexander Borowsky, UC Davis Cancer Center, USA

Maria Boucher, UNC Department of Pediatric Hematology Oncology, USA

Luke Boulter, MRC Human Genetics Unit, University of Edinburgh, UK

Angela Bowman, Washington University in St. Louis, USA

Salvatore Bozzaro, University of Turin, Italy

Thomas Brand, Imperial College London, UK

Cory Brayton, Johns Hopkins University, USA

Kendal Broadie, Vanderbilt University and Medical School, USA

Susan Brockerhoff, University of Washington, USA

Hannah Brunsdon, University of Edinburgh, UK

Robert Bryson-Richardson, Monash University, Australia

Alexa Burger, University of Colorado, USA

Felicity Burt, University of the Free State, South Africa

Ross Cagan, School of Cancer Sciences, University of Glasgow, UK

Michele Calder, University of Western Ontario, Canada

Guy Caldwell, University of Alabama, USA

Juan Calix, University of Alabama, Birmingham, USA

Brian Calvi, Indiana University Bloomington, USA

Katie Campbell, University of California, Los Angeles, USA

Giulia Campostrini, Leiden University Medical Center, the Netherlands

Changchun Cao, Nanjing Medical University, Nanjing First Hospital, China

Valeria Capra, IRCCS Giannina Gaslini Institute, Italy

Neil Carragher, University of Edinburgh, Western General Hospital, UK

Desroches Castan, INSERM, France

Margarida Castro Gomes, Mostowy Lab, London School of Hygiene and Tropical Medicine, UK

Christian Chabbert, Université Aix-Marseille, France

Mohamed Chahine, Laval University and CERVO Brain Research Centre, Canada

Yang Chai, University of Southern California, USA

Anob Chakrabarti, The Francis Crick Institute, UK

Karen Chang, University of Southern California, USA

Hsiao-Tuan Chao, Baylor College of Medicine, USA

Michael Cheeseman, Roslin Institute, UK

Ko-Fan Chen, University of Leicester, UK

Rui Chen, Baylor College of Medicine, USA

Huaiyong Chen, Tianjin University, China

Ke Cheng, North Carolina State University, USA

Wing Hoi Cheung, The Chinese University of Hong Kong, Hong Kong

Philippe Chevalier, Université Claude-Bernard Lyon 1, France

Yee Lian Chew, Flinders University, Australia

Kwang-Wook Choi, Korea Advanced Institute of Science and Technology, Republic of Korea

Ling-shiang Chuang, Icahn School of Medicine at Mount Sinai, USA

David Church, University of Oxford, UK

Steven Clapcote, University of Leeds, UK

Ben Clarke, The Francis Crick Institute, UK

Sebastian Clauss, Department of Cardiology, LMU Klinikum, Germany

David Clouthier, University of Colorado, USA

Robert Coffey, Vanderbilt University, USA

Christopher Colwell, University of California, Los Angeles, USA

Simon Conway, Indiana University, USA

Mark Cookson, National Institutes of Health, USA

Andrew Copp, Institute of Child Health, University College London, UK

Julia Cordero, University of Glasgow, UK

Martina Cornel, Amsterdam UMC, the Netherlands

Mark Cronan, Max Planck Institute for Infection Biology, Germany

Rachelle Crosbie, University of California, Los Angeles, USA

Merlin Crossley, UNSW Sydney, Australia

D. Kotresha, Davangere University, India

Andrea Daniel, Duke University Medical Center, USA

Tatyana Danyukova, University Medical Center Hamburg-Eppendorf, Germany

Arvin Dar, Icahn School of Medicine at Mount Sinai, USA

Lawrence David, Duke University School of Medicine, USA

Paolo De Coppi, Great Ormond Street Hospital, UK

Sofia de Oliveira, Albert Einstein College of Medicine, USA

Carmen de Sena Tomás, University of Lisbon, Portugal

Jordy Dekker, Erasmus MC, the Netherlands

Paul Delgado Olguin, The Hospital for Sick Children, Canada

Ihsan Demir, Klinikum rechts der Isar, Technical University of Munich, Germany

Jeroen den Hertog, Hubrecht Institute, the Netherlands

Qing Deng, Purdue University, USA

Rajendar Deora, Ohio State University, USA

Muriel Desbois, Seattle Children's Institute, USA

Michael Devine, The Francis Crick Institute, UK

Jordi Díaz-Manera, Newcastle University, UK

Luisa DiPietro, University of Illinois Chicago, USA

Nabil Djouder, Centro Nacional de Investigaciones Oncológicas, Spain

Leonard Dobens, University of Missouri–Kansas City, USA

Pedro Domingos, ITQB, Universidade NOVA de Lisboa, Portugal

Olivier Dorchies, University of Geneva, Switzerland

Richard Dorsky, University of Utah School of Medicine, USA

James Dowling, The Hospital for Sick Children, Canada

Ioannis Dragatsis, University of Tennessee, USA

Dongsheng Duan, University Missouri, USA

Nicole Dubois, Icahn School of Medicine at Mount Sinai, USA

Jessica Duis, Children's Hospital Colorado, USA

Philip Dunne, Queen's University Belfast, UK

Nicole Edwards, Cincinnati Children's Hospital Medical Center, USA

Lobna Elkhadragy, University of Illinois Chicago, USA

Olga Ermakova, Institute of Cell Biology and Neurobiology, Italy

Jeffrey Essner, Iowa State University, USA

Andrea Esteves, Universidade Estadual de Campinas, Brazil

Todd Evans, Weill Cornell Medicine, USA

Walid Fakhouri, University of Texas Health Science Center at Houston, USA

Christopher Fang-Yen, University of Pennsylvania, USA

Saranna Fanning, Harvard Medical School and Brigham and Women's Hospital, USA

Edward Farmer, University of Lausanne, Switzerland

Yi Feng, University of Edinburgh, UK

Miguel Ferreira, Institute for Research on Cancer and Aging, Nice, France

Silvia Finnemann, Fordham University, USA

Anthony Firulli, Indiana University School of Medicine, USA

Elizabeth Fisher, University College London, UK

Shannon Fisher, Boston University School of Medicine, USA

Sarah Fitzpatrick, Yale School of Medicine, USA

Edan Foley, University of Alberta, Canada

Antonella Forlino, University of Pavia, Italy

Ron Gaba, University of Illinois Hospital, USA

Davide Gabellini, IRCCS Ospedale San Raffaele, Italy

Gabriel Galea, UCL Great Ormond Street Institute of Child Health, UK

Rene Galindo, University of Texas Southwestern Medical Center, USA

Subramaniam Ganesh, Indian Institute of Technology Kanpur, India

Jose Garcia-Perez, University of Edinburgh, UK

Gaoxiang Ge, Shanghai Institutes for Biological Sciences, Chinese Academy of Sciences, China

Paola Ghezzi, IRCCS Ospendale San Raffaele, Italy

Pablo Giraudi, Fondazione Italiana Fegato, Italy

Diego Gómez-Nicola, University of Southampton, UK

Alfredo Gonzalez-Sulser, University of Edinburgh, UK

Margaret (Peggy) Goodell, Baylor College of Medicine, USA

June Goto, Cincinnati Children's Hospital Medical Center, USA

David Greaves, University of Oxford, UK

Rebecca Green, University of Pittsburgh, USA

Andrea Greiter-Wilke, Roche Pharma Research and Early Development, Switzerland

Brock Grill, University of Washington and Seattle Children's Research Institute, USA

Daniel Grimes, University of Oregon, USA

Yevgenya Grinblat, University of Wisconsin, USA

Valery Grinevich, Heidelberg University, Germany

Anna Grosberg, University of California, Irvine, USA

Alecia Gross, University of Alabama at Birmingham, USA

Miranda Grounds, University of Western Australia, Australia

Liubov Gushchina, Abigail Wexner Research Institute, Nationwide Children’s Hospital, USA

Kristina Haase, European Molecular Biology Laboratory, Spain

Melissa Haendel, University of Colorado Anschutz Medical Campus, USA

Melanie Haffner-Luntzer, University Medical Centre Ulm, Germany

Sassan Hafizi, University of Portsmouth, UK

René Hägerling, Charité – Universitätsmedizin Berlin, Germany

Xin Hailiang, Second Military Medical University, China

Mohammad Hajihosseini, University of East Anglia, UK

Nils Halberg, University of Bergen, Norway

Martina Hallegger, The Francis Crick Institute and University College London, UK

Penelope Hallett, McLean Hospital/Harvard Medical School, USA

Ross Hardison, Pennsylvania State University, USA

Melody Harper, Boston College, USA

Matthew Harris, Harvard Medical School, USA

Sneh Harsh, NYU School of Medicine, USA

Christine Hartmann, Universitätsklinikum Münster, Germany

Keiichi Hatakeyama, Shizuoka Cancer Center, Japan

Miep Helfrich, Institute of Medical Sciences, UK

Matthew Hemming, Dana-Farber Cancer Institute, USA

Deborah Henderson, Newcastle University, UK

Michael Henderson, Van Andel Research Institute, USA

Gretl Hendrickx, University of Antwerp, Belgium

Clarissa Henry, University of Maine, USA

Yann Herault, IGBMC-ICS, France

Adam Hill, Victor Chang Cardiac Research Institute, Australia

Susumu Hirabayashi, MRC London Institute of Medical Sciences, Imperial College London, UK

Larry Hoffman, David Geffen School of Medicine, USA

Ellen Hoffman, Yale University School of Medicine, USA

Shushu Huang, Yale University, USA

Alexander Hull, University College London, UK

John Hulleman, University of Texas Southwestern Medical Center, USA

Christy Hung, University College London, UK

Saskia Hurst, Max Planck Institute for Infection Biology, Germany

Tatsushi Igaki, Kyoto University, Japan

Khursheed Iqbal, University of Kansas Medical Center, USA

Kiyotake Ishikawa, Icahn School of Medicine at Mount Sinai, USA

Evgueni Ivakine, The Hospital for Sick Children, Toronto, Canada

Eri Iwasawa, Cincinnatti Children's Hospital Medical Center, USA

Hanna Jaaro-Peled, Johns Hopkins University, USA

Sujatha Jagannathan, University of Colorado Anschutz, USA

Vaibhao Janbandhu, Victor Chang Cardiac Research Institute, Australia

Thierry Jarde, Monash University, Australia

Parmjit Jat, University College London, UK

Brigid Jensen, Jefferson University, USA

Loydie Jerome-Majewska, McGill University, Canada

Dongyu Jia, Georgia Southern University, USA

Rulang Jiang, Cincinnati Children's Hospital, USA

Lin-Hua Jiang, University of Leeds, UK

Erin Jimenez, National Institutes of Health, USA

Peng Jin, Emory University School of Medicine, USA

Yongfeng Jin, Zhejiang University, China

Li Hua Jin, Northeast Forestry University, China

Mohit Kumar Jolly, Indian Institute of Science, India

Lesley Jones, Cardiff University, UK

James Jontes, Ohio State University, USA

Louise Jordon, University of Cambridge, UK

Diana Juriloff, University of British Columbia, Canada

Monica Justice, The Hospital for Sick Children, Canada

Peter Kang, University of Minnesota, USA

Gautam Kao, University of Gothenburg, Sweden

Swathi Karthikeyan, Stanford University, USA

Charles Kaufman, Washington University School of Medicine, USA

Yoshitaka Kawai, Kyoto University, Japan

Maria-Cristina Keightley, La Trobe University, Australia

Kate Keller, Oregon Health Sciences University, USA

Cheryl A. Keller, Eberly College of Science, USA

Sarada Ketharnathan, Childrens Hospital of Eastern Ontario, Canada

Benard Khor, Benaroya Research Institute, USA

Thomas Kidd, University of Nevada, Reno, USA

Kerri Kinghorn, University College London, UK

David Kirsch, Duke University, USA

Attila Kiss, Medical University of Vienna, Austria

Eddy Kizana, Faculty of Medicine and Health, University of Sydney, Australia

Christian Klämbt, University of Muenster, Germany

Susanne Klaus, German Institute of Human Nutrition, Germany

Pierre Klein, University College London/The Francis Crick Institute, UK

Nikolai Klymiuk, Technical University of Munich, Germany

Kim Kobar, University of Ottawa, Canada

Patryk Konieczny, Adam Mickiewicz University, Poznań, Poland

Kishore Kumar, University of Sydney, Australia

Kristen Kwan, University of Utah, USA

Nicole Lake, Yale University, USA

Robert Layfield, University of Nottingham Medical School, UK

Elisa Lazzari, University of Trieste, Italy

Je Chul Lee, Kyungpook National University, Republic of Korea

Helmar Lehmann, Uniklinik Köln, Germany

Harry Leitch, MRC London Institute of Medical Sciences, UK

Monkol Lek, Yale University, USA

Imre Lengyel, Queen’s University Belfast, UK

Cammie Lesser, Harvard University, USA

Patrick Lewis, Reading University, UK

Fiona Lewis-McDougall, Queen Mary University of London, UK

Yun Li, University of Toronto, Canada

Leanne Li, The Francis Crick Institute, UK

Shupeng Li, Peking University, China

Jiada Li, Central South University China, China

Eric Liao, Children's Hospital of Philadelphia, USA

Shane Liddelow, NYU Grossman School of Medicine, USA

Ching-Ling Lien, University of Southern California, USA

Graham Lieschke, Australian Regenerative Medicine Institute, Australia

Gabriele Lignani, University College London, UK

Chris Link, University of Colorado, USA

Robert Lipinski, University of Wisconsin-Madison, USA

Jonathan Lippiat, University of Leeds, UK

Junlai Liu, University of California San Diego, USA

Naikui Liu, Indiana University School of Medicine, USA

Hongbing Liu, Tulane, USA

Clifford Lowell, University of California, San Francisco, USA

Jie Lu, Affiliated Hospital of Qingdao University, China

Wei-Yu Lu, University of Edinburgh, UK

Alejandro Lucía, Universidad Europea de Madrid, Spain

Raphaelle Luisier, Idiap Research Institute, Switzerland

Yilun Ma, Weill Cornell Medicine, USA

Thomas MacDonald, Barts and The London School of Medicine and Dentistry, UK

Bhavani Madakashira, NYU Abu Dhabi, United Arab Emirates

Khalid Mahmood, University of Melbourne, Australia

Bilal Malik, UCL Queen Square Institute of Neurology, UK

Saira Malik, Michigan State University, USA

Giovanni Manfredi, Weill Cornell Medicine, USA

Eirini Maniou, University College London, UK

Roope Mannikko, University College London, UK

Abed Mansour, Hebrew University of Jerusalem, Israel

Maria Carolina Marchetto, University of California San Diego, USA

Henry Martin, University College London, UK

Anthony Martinelli, University of Cambridge, UK

Disease Models & Mechanisms • Supplementary information

Spyros Marvopoulos, Icahn School of Medicine at Mount Sinai, USA

John Mason, University of Edinburgh, UK

Thomas Massey, Cardiff University, UK

Yutaka Matsubayashi, Bournemouth University, UK

Lisa Maves, Seattle Children's Research Institute, USA

Jeffrey McArthur, Victor Chang Cardiac Research Institute, Australia

Robert McDonald, University of Lethbridge, Canada

Ian McGough, Babraham Institute, UK

Kyle McLean, Massachusetts Institute of Technology, USA

Hayley McLoughlin, University of Michigan, USA

Sarah McMenamin, Boston College, USA

Rosaria Meccariello, Parthenope University of Naples, Italy

Sigolène Meilhac, Institut Imagine, Institut Pasteur, France

Miriam Meisler, University of Michigan, USA

Aswin Menke, TNO Triskelion Zeist, the Netherlands

Germana Meroni, University of Trieste, Italy

Amy Merrill-Brugger, University of Southern California, USA

Pablo Meyer, IBM Research, USA

Gretchen Meyer, Washington University in St. Louis, USA

David Meyerholz, University of Iowa, USA

Marja Mikkola, University of Helsinki, Finland

Marco Milan, IRB Barcelona, Spain

Rachel Miller, McGovern Medical School, USA

Crispin Miller, CRUK Beatson Institute, UK

Andrew Miller, University of Wisconsin-Madison, USA

Berge Minassian, University of Texas Southwestern Medical Center, USA

James Minchin, University of Edinburgh, UK

Mayssa Mokalled, Washington University School of Medicine, USA

Sally Moody, George Washington University, USA

Roy Morello, University of Arkansas for Medical Sciences, USA

Orson Moritz, University of British Columbia, USA

Enrico Moro, University of Padova, Italy

Heather Mortiboys, University of Sheffield, UK

Jennifer Morton, The Beatson Institute, UK

Christian Mosimann, University of Colorado School of Medicine, Anschutz Medical Campus, USA

Serge Mostowy, London School of Hygiene and Tropical Medicine, UK

Christopher Moxon, University of Glasgow, UK

Akankshi Munjal, Duke University, USA

Daniel Murphy, University of Glasgow, UK

Laura Musselman, Binghamton University, USA

Kevin Myant, University of Edinburgh, UK

Sandesh Nagamani, Baylor College of Medicine, USA

Hiroshi Nakagawa, Columbia University, USA

Kihoon Nam, University of Missouri-Columbia, USA

Nawazish Naqvi, Emory University, USA

James Nathan, University of Cambridge, UK

Salvatore Nesci, Università di Bologna, Italy

Sherylanne Newton, University College London, UK

Teresa Niccoli, University College London, UK

Laurence Nieto, Centre de Recherches en Cancérologie de Toulouse, France

Clévio Nóbrega, University of Algarve, Portugal

Kerby Oberg, Loma Linda University, USA

Natasha O'Brown, Harvard Medical School, USA

Kazuhide Okuda, Peter MacCallum Cancer Centre, Australia

Menno Oudhoff, Norwegian University of Science and Technology, Norway

Raghu Padinjat, National Centre for Biological Sciences, India

Damon Page, University of Florida Scripps Biomedical Research, USA

Koustav Pal, The Francis Crick Institute, UK

James Palis, University of Rochester, USA

Yuchin Pan, Virginia Tech, USA

Pan Pan, Peking Union Medical College, China

Alex Parker, CRCHUM, Université de Montréal, Canada

Linda Partridge, University College London, UK

E. Elizabeth Paton, University of Edinburgh, UK

Ralph Patrick, Victor Chang Cardiac Research Institute, Australia

Andrew Patterson, Pennsylvania State University, USA

Erwin Pauws, University College London, UK

David Pearce, Sanford Research, USA

Claire Pearson, University of Oxford, UK

Jason Peart, Griffith University, Australia

Carmen Pedraza, Universidad de Malaga, Spain

Caroline Pellet-Many, Royal Veterinary College, UK

Norbert Perrimon, Harvard Medical School, USA

Randall Peterson, University of Utah, USA

Frank Pfrieger, Centre National de la Recherche Scientifique, France

Shubhangi Pingle, Regional Occupational Health Center (Southern), National Institute of Occupational Health, India

Angelo Poletti, University of Milan, Italy

Steve Pollard, University of Edinburgh, UK

Alexander Poltorak, Tufts University School of Medicine, USA

Enzo Porrello, Murdoch Children's Research Institute, Australia

Andrew Preston, University of Bath, UK

William Pyle, University of Guelph, Canada

Satyanarayana Rachagani, University of Nebraska Medical Center, USA

Sunniyat Rahman, University College London, UK

Sara Rashkin, St Judes Research Hospital, USA

Martina Rauner, Technische Universität Dresden, Germany

Andrea Rauschmayer, Baylor College of Medicine, USA

Robert Rawson, Western Governors University, USA

Keisha Ray, University of Texas Health Science Center at Houston, USA

Bruno Reichart, LMU Munich, Germany

Mindong Ren, NYU Grossman School of Medicine, USA

Jason Rihel, University College London, UK

Paul Riley, University of Oxford, UK

Sara Risseeuw, UMC Utrecht, the Netherlands

Karine Rizzoti, The Francis Crick Institute, UK

Ed Roberts, CRUK Beatson Institute, UK

Peter Robinson, Charite Universitätsmedizin Berlin, Germany

John Robinson, University of Rhode Island, USA

Adela Rodriguez-Romero, Universidad Nacional Autonoma de Mexico, Mexico

Maria Rohm, Helmholtz Zentrum München, Germany

Randall Roper, Indiana University Perdue University Indianapolis, USA

Jessica Rosati, Fondazione IRCCS Casa Sollievo della Sofferenza, Italy

Emily Rosowski, Clemson University, USA

M. A. Rüegg, University of Basel, Switzerland

Amy Rumora, Columbia University, USA

B. Mohd Sabri, University of Malaysia, Malaysia

Kirsten Sadler Edepli, New York University Abu Dhabi, United Arab Emirates

Takuya Sakaguchi, Cleveland Clinic, USA

Paolo Salomoni, German Center for Neurodegenerative Diseases, Germany

Charles Sanders, Vanderbilt University, USA

Leslie Sanderson, Erasmus Medical Center, the Netherlands

Owen Sansom, The Beatson Institute for Cancer Research, UK

Sumana Sanyal, Sir William Dunn School of Pathology, UK

Hiroki Sasaguri, RIKEN Center for Brain Science, Japan

J. D. Sauer, University of Wisconsin-Madison, USA

Stephen Sawcer, University of Cambridge, UK

Peter Scambler, University College London, UK

Angelika Schnieke, Technical University of Munich, Germany

Lynn Schriml, University of Maryland, USA

Ben Schumann, Imperial College London, UK

Brian Schutte, Michigan State University, USA

Jens Schwamborn, University of Luxembourg, Luxembourg

Ian Scott, Sick Kids Research Institute, Canada

Christoph Seiler, Children's Hospital of Philadelphia, USA

Bhuvaneish Selvaraj, University of Edinburgh, UK

Robert Semple, University of Edinburgh, UK

Jane Seto, Murdoch Children's Research Institute, Australia

Claudio Sette, University Cattolica del Sacro Cuore, Italy

Ji Shanming, IBMC Strasbourg, France

Yojet Sharma, University of Mumbai, India

Jordan Shavit, University of Michigan, USA

Dana Shaw, Washington University in St. Louis, USA

Simone Shen, University of Wisconsin, USA

Hongying Shen, Yale University, USA

Celia Shiau, University of North Carolina at Chapel Hill, USA

Yuji Shiba, Shinshu University, Japan

Ming-Sing Si, David Geffen School of Medicine, University of California, Los Angeles, USA

Chris Sibley, University of Edinburgh, UK

Julie Siegenthaler, University of Colorado, USA

Dirk Sieger, University of Edinburgh, UK

Daria Siekhaus, Institute of Science and Technology Austria, Austria

Jimena Sierralta, Universidad de Chile, Chile

Dina Simkin, Northwestern University, USA

Filipa Simões, University of Oxford, UK

David Sims, University of Oxford, UK

James Sleigh, University College London, UK

Erica Sloan, Monash University, Australia

Kourtney Sloan, University of Indianapolis, USA

Eric Small, University of Rochester Medical Center, USA

Kelly Smith, University of Melbourne, Australia

Kenneth Smith, University College London, UK

Juhoon So, University of Pittsburgh, USA

Veronica Uribe Sokolov, University of Melbourne, Australia

Guisheng Song, University of Minnesota, USA

Masahiro Sonoshita, Institute for Genetic Medicine, Hokkaido University, Japan

Tomokazu Souma, Duke University, USA

Michelle Southard-Smith, Vanderbilt University Medical Center, USA

Ramanathan Sowdhamini, National Centre for Biological Sciences, TIFR, India

Malte Spielmann, University Medical Center Schleswig-Holstein, Germany

Erin Spiller, Heidelberg University, Germany

Alessio Squassina, University of Cagliari, Italy

Katja Steiger, Technical University of Munich, Germany

Edward Stephens, University of Kansas Medical Center, USA

Knut Stieger, University of Giessen, Germany

Rolf Stottmann, Nationwide Children's Hospital, USA

Helen Strutt, University of Sheffield, UK

Tin Tin Su, University of Colorado Boulder, USA

Longxiang Su, Peking Union Medical College Hospital, China

Hidetaka Suga, Nagoya University Graduate School of Medicine, Japan

Andreas Suhrbier, Australian Infectious Diseases Research Centre, Brisbane, Australia

Alyson Sujkowski, Wayne State University, USA

Kate Sutherland, Walter and Eliza Hall Institute, Australia

Larry Suva, Texas A&M University, USA

Fran Sverdrup, Saint Louis University School of Medicine, USA

Katherine Swenson-Fields, University of Kansas Medical, USA

Ramizah Syahirah, Purdue University, USA

Doaa Taha, The Francis Crick Institute, UK

Kazuo Takayama, Kyoto University, Japan

Patrick Tang, Chinese University of Hong Kong, Hong Kong

Marc Tatar, Brown University, USA

Irmgard Tegeder, Goethe-University Hospital, Frankfurt, Germany

Jason Tennessen, Indiana University, USA

David Thomson, Brigham Young University, USA

Summer Thyme, University of Alabama at Birmingham, USA

Malte Tiburcy, University Medical Center Göttingen, Germany

Gavin Tjin, St. Vincent's Institute of Medical Research, Australia

David Tobin, Duke University Medical Center, USA

Sokol Todi, Wayne State University, USA

Anna Törnqvist, University of Gothenburg, Sweden

Andrew Tosolini, University College London, UK

Paul Trainor, Stowers Institute for Medical Research, USA

Eirini Trompouki, University of Cote D'Azur, France

Michael Tsang, University of Pittsburgh, USA

Abigail Tucker, King's College London, UK

Suzanne Turner, University of Cambridge, UK

Luke Tweedy, The Beatson Institute, University of Glasgow, UK

Victor Tybulewicz, The Francis Crick Institute, UK

Giulia Tyzack, The Francis Crick Institute, UK

Prech Uapinyoying, National Institutes of Health, USA

Patricia Vaduva, Endocrinologue et Metabolisme, France

Raphael Valdivia, Duke University School of Medicine, USA

Julie Van De Weghe, University of Oklahoma Health Sciences Center, USA

Sjoerd van Wijk, Goethe University Frankfurt, Germany

Jamie Vandenberg, Victor Chang Cardiac Research Institute, Australia

Rajanikanth Vangipurapu, Saint Louis University School of Medicine, USA

José Vargas-Muñiz, Southern Illinois University, USA

Neil Vargesson, University of Aberdeen, UK

Esther Verheyen, Simon Fraser University, Canada

John Noel Viana, Australian National University, Australia

Jeanette Villanueva, Victor Chang Cardiac Research Institute, Australia

Francesc Villarroya, Institut de Biomedicina, Universitat de Barcelona, Spain

Ajoy Vincent, The Hospital for Sick Children, Canada

Douglas Vollrath, Stanford University School of Medicine, USA

Raimund Wagener, Universität zu Köln, Germany

Yogesh Wairkar, University of Texas Medical Branch, USA

James Walker, Mass General Hospital, USA

Lucas Waltzer, GReD, CNRS, France

Hui-Li Wang, Hefei University of Technology, China

Zhi-Chao Wang, Shanghai Jiao Tong University School of Medicine, China

Timothy Wang, Columbia University, USA

Jianhai Wang, Nanjing Tech University, China

Brandon Warren, University of Florida, USA

John Warrick, University Richmond, USA

Nicole Weaver, University of Notre Dame, USA

Michael Weinberger, University of Oxford, UK

Mitchell Weiss, St. Jude Childrens Research Hospital, USA

Zilong Wen, Hong Kong University of Science and Technology, China

Robert Wessells, Wayne State University, USA

David Westaway, University of Alberta, Canada

Bart Westendorp, Utrecht University, Faculty of Veterinary Medicine, the Netherlands

Robert Wheeler, University of Maine, USA

Richard White, Sloan Kettering Institute, USA

Richard White, University of Oxford, UK

Paul Whiting, University College London, UK

Trevor Williams, University of Colorado Denver, USA

Rebecca Wingert, University of Notre Dame, USA

Sophie Wiszniak, University of South Australia, Australia

Eckhard Wolf, Ludwig-Maximilians-Universität München, Germany

Mingfu Wu, University of Houston, USA

Nan Wu, Indiana University School of Medicine, USA

Anping Xia, Stanford University, USA

Zhuo Xing, Roswell Park Cancer Institute, USA

Hui Xiong, Peking University First Hospital, China

Yojiro Yamanaka, McGill University, Canada

Peixin Yang, University of Maryland School of Medicine, USA

Jing-Ruey Yeh, Massachusetts General Hospital/Harvard Medical School, USA

Marielle Yohe, National Cancer Institute, USA

Sa Kan Yoo, RIKEN Center for Biosystems Dynamics Research, Japan

Y. Yu, Roswell Park Cancer Institute, USA

Xinge Yu, University of Michigan, USA

Ryan Yuen, The Hospital for Sick Children, Canada

Sarah Zankar, The Ottawa Hospital, Canada

Michael Zech, Helmholtz Zentrum München, Germany

Jin Zhang, Washington University School of Medicine, USA

Jingyuan Zhang, Harvard Medical School/Boston Children Hospital, USA

Yuan Zhu, Children’s National Hospital, USA

Wenhan Zhu, Vanderbilt University Medical Center, USA

Leonard Zon, Howard Hughes Medical Institute, USA

Clara Zourray, University College London, UK
